# Autoimmune disease associated IFIH1 single nucleotide polymorphism related with IL-18 serum levels in Chinese systemic lupus erythematosus patients

**DOI:** 10.1038/s41598-018-27782-7

**Published:** 2018-06-21

**Authors:** Junlong Zhang, Xinle Liu, Yanming Meng, Hengxu Wu, Yongkang Wu, Bin Yang, Lanlan Wang

**Affiliations:** Department of Laboratory Medicine, West China Hospital, Sichuan University, Chengdu, Sichuan Province China

## Abstract

Systemic lupus erythematosus (SLE) has heterogeneous clinical manifestations. IFIH1 (interferon induced with helicase C domain 1) as one of antiviral helicase genes mediating type I interferon production, plays an essential role in the pathogenesis of SLE. The gene variants in IFIH1 could abnormally activate antiviral defenses and increased type I interferon signaling. The present study aimed to validate associations between single nucleotide polymorphisms (SNP) in IFIH1 and the pathogenesis of SLE. In total, rs1990760, rs3747517 and rs10930046 in IFIH1 are genotyped in 400 SLE patients and 659 health controls in Chinese cohort by an improved multiplex ligation detection reaction (iMLDR) technique. Significant associations were observed between alleles of IFIH1 (rs1990760 C > T, P = 0.005, OR = 1.36, 95%CI = 1.10–1.69; rs3747517 T > C, P = 0.004, OR = 1.31, 95%CI = 1.09–1.58, respectively) and SLE susceptibility. IFIH1 rs1990760 TT genotype carriers had lower serum levels of IL-18 (P < 0.001) and granzyme B (P < 0.001) than CC and CT genotype carriers. IFIH1 rs1990760 CT genotype carriers had higher anti-dsDNA–positive than CC and TT genotype carriers. In conclusion, IFIH1 polymorphisms (rs1990760 and rs3747517) were associated with SLE susceptibility and rs1990760 risk T allele related with IL-18 and granzyme B serum levels in SLE patients.

## Introduction

Systemic lupus erythematosus (SLE) is a systemic multiorgan autoimmune disease which is characterized by chronic inflammation mediated by diversified immunologic factors. The underlying biological mechanisms in the pathogenesis of SLE remain uncertain^1–3^. SLE has been shown to have inappropriately activated antiviral defenses and increased IFN-α signaling^4–6^. Accordingly, as one of the antiviral helicase genes mediating type I interferon production, IFIH1 (interferon induced with helicase C domain 1) may play an essential role both in the pathogenesis and progression of SLE^7^. As a member of the retinoic acid-inducible gene I (RIG-I) -like receptor family, the IFIH1 protein also known as MDA5 (melanoma differentiation-associated protein 5), is highly upregulated in activated immune cells in response to type I interferon and is expressed at lower levels in resting immune cells and other tissues, including pancreatic islets^8^.

IFIH1 encodes an interferon-inducible RNA helicase that, together with RIG-I, functions as an early-response cytoplasmic double-stranded RNA(dsRNA) sensor to detect viral infections and then elicit antiviral responses through the activation of the interferon-regulatory factors (IRF3) and nuclear factor kappa B (NF-κB) transcription factors^8–10^. Previous GWAS on IFIH1 identified the association between few SNPs in this gene with the risk of various autoimmune diseases including type 1 diabetes (T1DM), multiple sclerosis, psoriasis, selective IgA deficiency, dilated cardiomyopathy and SLE^11,12^. IFIH1 had been proved to be one of the genes which play an important role in the pathogenesis of the condition of SLE based on a large size of sampling in England^12^. Variation at rs1990760 in IFIH1 can influence gene expression^13^. The T risk allele at rs1990760 can increase the expression of IFIH1 gene and regulate the production of type I IFN and various proinflammatory cytokines which has great influence in the pathogenesis and severity of SLE^14–16^. Evidence suggests that rs1990760 may have a trend of association with reduced expression of IFIH1 with the protection in T1DM^15^. Patients carrying TT genotype at rs1990760 had significantly higher IFIH1 transcript levels compared to individuals carrying non-risk allele homozygous^12^. Based on the previous studies, we hypothesize that IFIH1 variants (rs1990760, rs3747517 and rs10930046) might interrupt pro-inflammatory responses and affect the cytokines in SLE patients. And as expected, we found that these SNPs had a stronger magnitude of increased risk for SLE and associated with serum levels of IL-18 and granzyme B.

## Materials and Methods

### Patients and protocol

There were a total of 1059 participants, including 400 SLE cases and 659 gender-and age-matched health controls were enrolled in this study from 2014 to 2016 in West China Hospital. All the patients hospitalized without drug-induced SLE, diagnosed as SLE in compliance with the American College of Rheumatology classification criteria for SLE revised in 1997^17^. They were all in the active stage with SLE disease activity index (SLEDAI) > 4. The main clinical manifestations such as arthritis, proteinuria, malar rash, pleuritis, pericarditis, neurological disorder, haematological disorder and lupus nephritis were obtained retrospectively by reviewing patients’ history records. The healthy controls should meet these inclusion criteria: without any chronic, endemic infectious or any types of autoimmune disorders and with normal physical examination and blood tests. This study conducted in accordance with the 1975 Declaration of Helsinki and was approved by the Ethics Committee of West China Hospital. Informed consent was obtained from all individual participants included in the study.

### Cytokines and autoantibodies measurement

155 SLE patients were randomly selected for testing the serum levels of IL-18 and granzyme B. IL-18 and granzyme B were determined through R&D Human Inflammation Assays. 400 SLE patients were detected with autoantibodies(ANA, anti-dsDNA and anti-Sm) by using ANA, anti-dsDNA and ENA reagent kits from Euroimmun (Euroimmun, Germany), respectively. All the tests were performed according to manufacturers’ instruction. Serum IL-18, granzyme B levels and autoantibodies of SLE patients were assessed at the same time during the clinical examination.

### IFIH1 polymorphism genotyping and LD evaluation

Genomic DNA was isolated from 200 μl of EDTA noncoagulated blood samples from each subjects using QuickGene DNA whole blood kit S (QuickGene, FujiFilm, Japan). The SNP genotyping was performed using an improved multiplex ligation detection reaction (iMLDR) technique (Genesky Biotechnologies Inc., Shanghai, China). The PCR programme for reactions was 95 °C for 2 min, then 11 cycles × (94 °C for 20 s, 65 °C–0.5 °C/cycle for 40 s, and 72 °C for 1 min), followed by 24 cycles of 94 °C for 20 s, 59 °C for 30 s, and 72 °C for 1.5 min, then 72 °C for 2 min, and 4 °C thereafter. The ligation cycling programme was 95 °C for 2 min; 38 cycles of 1 min at 94 °C and 4 min at 56 °C; hold at 4 °C. 0.5 μl of ligation product was loaded into the ABI 3730XL and the initial data were analysed by GeneMapper 4.1 (AppliedBiosystems, Foster City, CA USA). A random sample accounting for approximately 10% of the total DNA samples was directly sequenced to confirm the iMLDR results. Linkage disequilibrium evaluation was conducted by the Haploview software (version 4.2)^18^.

### Statistical analysis

Statistical power was calculated using a software called “PS: Power and Sample size Calculation” (http://biostat.mc.vanderbilt.edu/wiki/Main/PowerSampleSize). Then the Hardy-Weinberg equilibrium (HWE) was evaluated for each polymorphism independently. We used Mean ± SD, median and interquartile to describe the continuous variables with normal and skewed distribution, respectively. To compare demographic and clinical data, we used Kruskal-Wallis test. Allele comparisons between patients and health controls were analyzed by pearson’s chi-square test. The odds ratio (OR) and 95% Confidence Interval (CI) was assessed to show the relationship between SNPs and SLE susceptibility. We compared the subjects from two groups: allelic frequency distribution of the two groups (When we use A as the major allele, B as the minor allele, dominant model is AB + BB versus AA); recessive model is BB versus AA + AB. All statistical analyses is done by Statistical Package for the Social Sciences (SPSS, SPSS Inc., Chicago, IL, USA), version 20.0. A two-sided P value <0.05 was considered to be statistically significant.

## Results

### Demographic characteristics of the study population

The general characteristics and primary demographic for all enrolled participates including 400 SLE patients and 659 health controls were summarized in Table 1. There were no significant differences in age and gender of the study (P = 0.586 and 0.848, respectively), with the mean age being 36.33 in SLE patients and 36.76 in health controls. The average disease duration of SLE patients was 36.00 (5.25–84.00) months and the average SLEDAI score was 15.51 ± 7.54. Among the SLE patients enrolled in this study, the prevalence of ANA, anti-dsDNA and anti-Sm were 96.50%, 65.00% and 43.75%, respectively; while the average serum C3 concentration was 0.51 ± 0.23 g/L, and the average C4 was 0.11 ± 0.06 g/L. In addition, positive frequencies of arthritis, proteinuria, malar rash, pleuritis, pericarditis, neurological disorder, haematological disorder and lupus nephritis were 25.75%, 85.00%, 39.75%, 31.25%, 31.25%, 11.50%, 32.25% and 63.50%, respectively.

**Table 1:** Demographics and clinical characteristics of subjects.

Characteristics	SLE	Control	P-value
Number of subjects	400	659	—
Age, mean ± SD (years)	36.33 ± 12.99	36.76 ± 11.40	0.586
Male (%)/Female (%)	41 (10.25)/359 (89.75)	70 (10.62)/589(89.38)	0.848
Disease duration (months)	36.00 (5.25–84.00)	—	—
SLEDAI, mean ± SD	15.51 ± 7.54	—	—
Arthritis (N(%))	103 (25.75%)	—	—
Proteinuria (N(%))	340 (85.00%)	—	—
Malar rash (N(%))	159 (39.75%)	—	—
Pleuritis (N(%))	125 (31.25%)	—	—
Pericarditis (N(%))	125 (31.25%)	—	—
Neurological disorder(N(%))	46 (11.50%)	—	—
Haematological disorder (N(%))	129 (32.25%)	—	—
Lupus nephritis (N(%))	254 (63.50%)	—	—
ANA-positive (N(%))	386 (96.50%)	—	—
dsDNA-positive (N(%))	260 (65.00%)	—	—
Sm-positive (N(%))	175 (43.75%)	—	—

### Polymorphisms association with SLE susceptibility in Chinese Cohort

Genotype and allele frequencies of IFIH1 SNPs (rs1990760, rs3747517 and rs10930046) in SLE patients and health subjects are depicted in Table 2. Significant differences in the allelic distributions at rs1990760 C > T (P = 0.005, statistical power = 0.816) and rs3747517 T > C (P = 0.004, statistical power = 0.823) between SLE group and healthy controls were observed. However, no significant differences among the variant frequencies at rs10930046 were found between SLE patients and health controls. We observed that CT genotype at rs1990760 was associated with SLE susceptibility (P = 0.006, OR = 1.46, 95%CI = 1.12–1.90; dominant model: P = 0.004, OR = 1.47, 95%CI = 1.13–1.90) (Table 2).

**Table 2:** Genotype and allele distributions of IFIH1 gene polymorphisms and associations with SLE.

SNPs	Model	Genotype	SLE		Controls		OR (95%CI)	*P-* value
			(n = 400)		(n = 659)			
			N	Frequency	N	Frequency		
rs1990760 C > T		CC	236	59.00%	447	67.83%	1	—
		CT	147	36.75%	191	28.98%	1.46 (1.12–1.90)	**0.006**
		TT	17	4.25%	21	3.19%	1.53 (0.79–2.96)	0.200
	Dominant	TT + CT/CC	—	69.49%	—	47.43%	1.47 (1.13–1.90)	**0.004**
	Recessive	TT/CT + CC	—	4.44%	—	3.29%	1.35 (0.70–2.59)	0.367
	Allelic	C	619	77.38%	1085	82.32%	1	—
		T	181	22.63%	233	17.68%	1.36 (1.10–1.69)	**0.005**
rs3747517 T > C		TT	161	40.25%	331	50.23%	1	—
		CT	193	48.25%	265	40.21%	1.50 (1.15–1.95)	**0.003**
		CC	46	11.50%	63	9.56%	1.50 (0.98–2.29)	0.060
	Dominant	CC + CT/TT	—	148.45%	—	99.09%	1.50 (1.17–1.93)	**0.002**
	Recessive	CC/CT + TT	—	12.99%	—	10.57%	1.23 (0.82–1.84)	0.314
	Allelic	T	515	64.38%	927	70.33%	1	—
		C	285	35.63%	391	29.67%	1.31 (1.09–1.58)	**0.004**
rs10930046 T > C		TT	298	74.50%	512	77.69%	1	—
		CT	98	24.50%	140	21.24%	1.20 (0.90–1.62)	0.220
		CC	4	1.00%	7	1.06%	0.98 (0.29–3.38)	0.977
	Dominant	CC + CT/TT	—	34.23%	—	28.71%	1.19 (0.89–1.59)	0.235
	Recessive	CC/CT + TT	—	1.01%	—	1.07%	0.94 (0.27–3.23)	0.923
	Allelic	T	694	86.75%	1164	88.32%	1	—
		C	106	13.25%	154	11.68%	1.15 (0.89–1.50)	0.287

### Polymorphisms association with serologic profile of SLE patients

**Figure 1:**
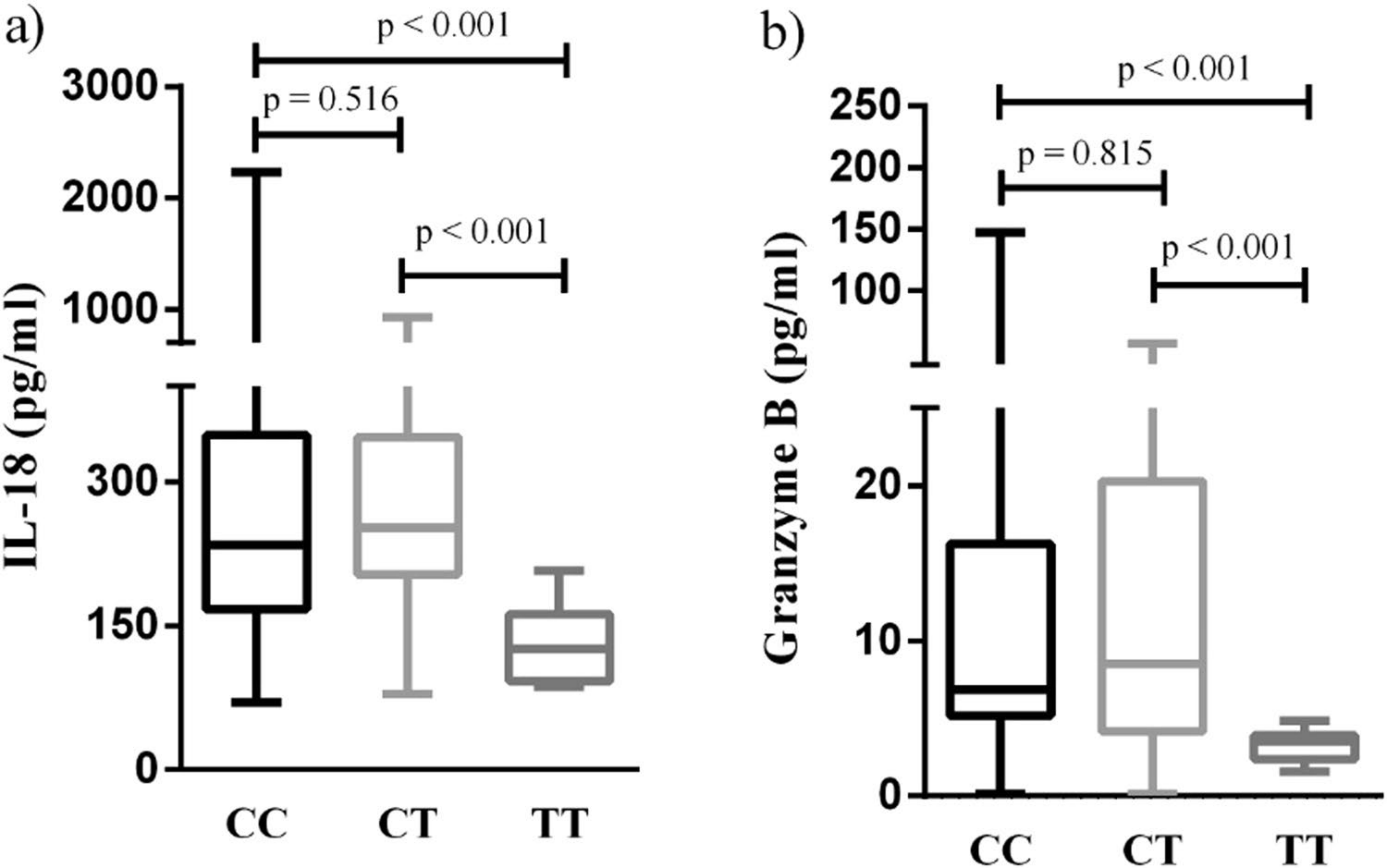
Association of IFIH1 rs1990760 genotypes with IL-18 and granzyme B serum levels. Serologic profile of SLE patients with different genotype of IFIH1 from 155 SLE patients (84 CC, 54 CT and 17 TT genotype for rs1990760). TT genotype carriers at rs1990760 in IFIH1 had lower serum levels of IL-18 and granzyme B than the CC and CT genotype carriers (P < 0.001, respectively). (**a**) IL-18 (pg/ml): CC genotype 234.13 (167.07,349.07), CT genotype 252.51 (203.74,347.16) and TT genotype 125.89 (92.33,162.06) for rs1990760, respectively. (**b**) Granzyme B (pg/ml): CC genotype 6.85 (5.19,16.24), CT genotype 8.49 (4.15,20.30) and TT genotype 3.53 (2.37,3.92) for rs1990760, respectively. The results represent the median and interquartile of IL-18 and granzyme B levels.

Results of the influence of the rs1990760 polymorphisms on serologic profile of SLE patients are illustrated in Fig. 1. The levels of IL-18 and granzyme B in serum had significant difference in SLE patients with different genotypes from 155 SLE patients (84 CC, 54 CT and 17 TT genotype for rs1990760). TT genotype carriers at rs1990760 in IFIH1 had lower levels of IL-18 and granzyme B than the CC and CT genotype carriers (P < 0.001, respectively). However, no significant differences between the variants (rs3747517 and rs10930046) and levels of IL-18 and granzyme B were found in SLE patients.

### Polymorphisms association with SLE autoantibody in Chinese Cohort

**Figure 2:**
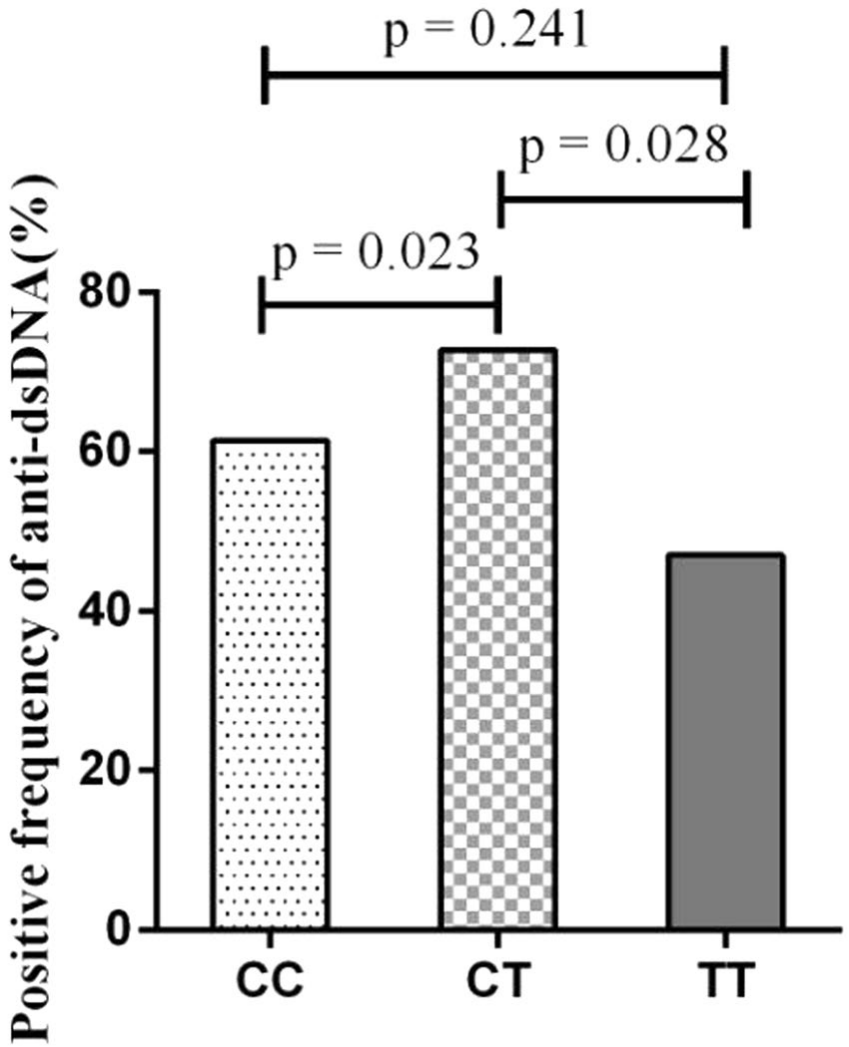
Association between rs1990760 genotypes and positive frequency of anti-dsDNA autoantibody in SLE patients. The positive frequency of anti-dsDNA with CC (61.44%), CT (72.79%) and TT (47.06%) genotypes of rs1990760 in SLE patients (CT vs CC, P = 0.023; CT vs TT, P = 0.028, respectively).

The SLE patients with CT genotype at rs1990760 had significant increased frequency of the positive anti-dsDNA autoantibody in Fig. 2. The positive frequency of anti-dsDNA with CT(72.79%) genotype compared with CC(61.44%) or TT(47.06%) genotype patients at rs1990760 (CT vs CC, P = 0.023; CT vs TT, P = 0.028, respectively) in SLE patients.

## Discussion

Our study first focused on the associations between IFIH1 variants (rs1990760, rs3747517 and rs10930046) and SLE susceptibility and the serum levels of inflammatory markers (IL-18 and granzyme B) in Chinese SLE patients.

SLE is a multisystem autoimmune disease characterized by the loss of self-tolerance^19^. Type I IFN and proinflammatory cytokines have a great influence on the pathogenesis and severity of SLE^14–16^. IFIH1 (also known as MDA5) senses viral RNA and helps to induce transcription of type I IFN and IFN-induced genes when activated^16^. Through activating transcription factors such as IRF3, IRF7 and NF-κB, IFIH1 could induce IFN-α/β and interferon-inducible genes to regulate the production of type I IFN and various proinflammatory cytokines^7^. In SLE patients, mutations in IFIH1 have been found in different cohorts^14,20,21^. The results of meta-analysis revealed significant association between the IFIH1 rs1990760 polymorphism and autoimmune diseases including T1DM and SLE^22^. Several studies have shown that the autoimmune-disease-associated allele of IFIH1 (rs1990760 T, 946 T) is likely to be a gain-of-function variation. The rs1990760 T risk allele was identified to cause a substantial increase in SLE susceptibility in different populations (including Chinese population)^14,20,21,23^, with increased expression of IFIH1^12,13,15^. The major allele at IFIH1 rs10930046 associated with increased risk of SLE in African-Americans^7^. In regard to IFIH1 rs3747517, which was embedded within an activator protein 1 (AP-1) binding site, no significant relation between rs3747517 and SLE susceptibility was observed in patients from Brazil^20^. However, ethnic differences have been seen in the allele distributions of both rs1990760 and rs3747517 in Europeans, Asians and Africans^20,24,25^.

**Figure 3:**
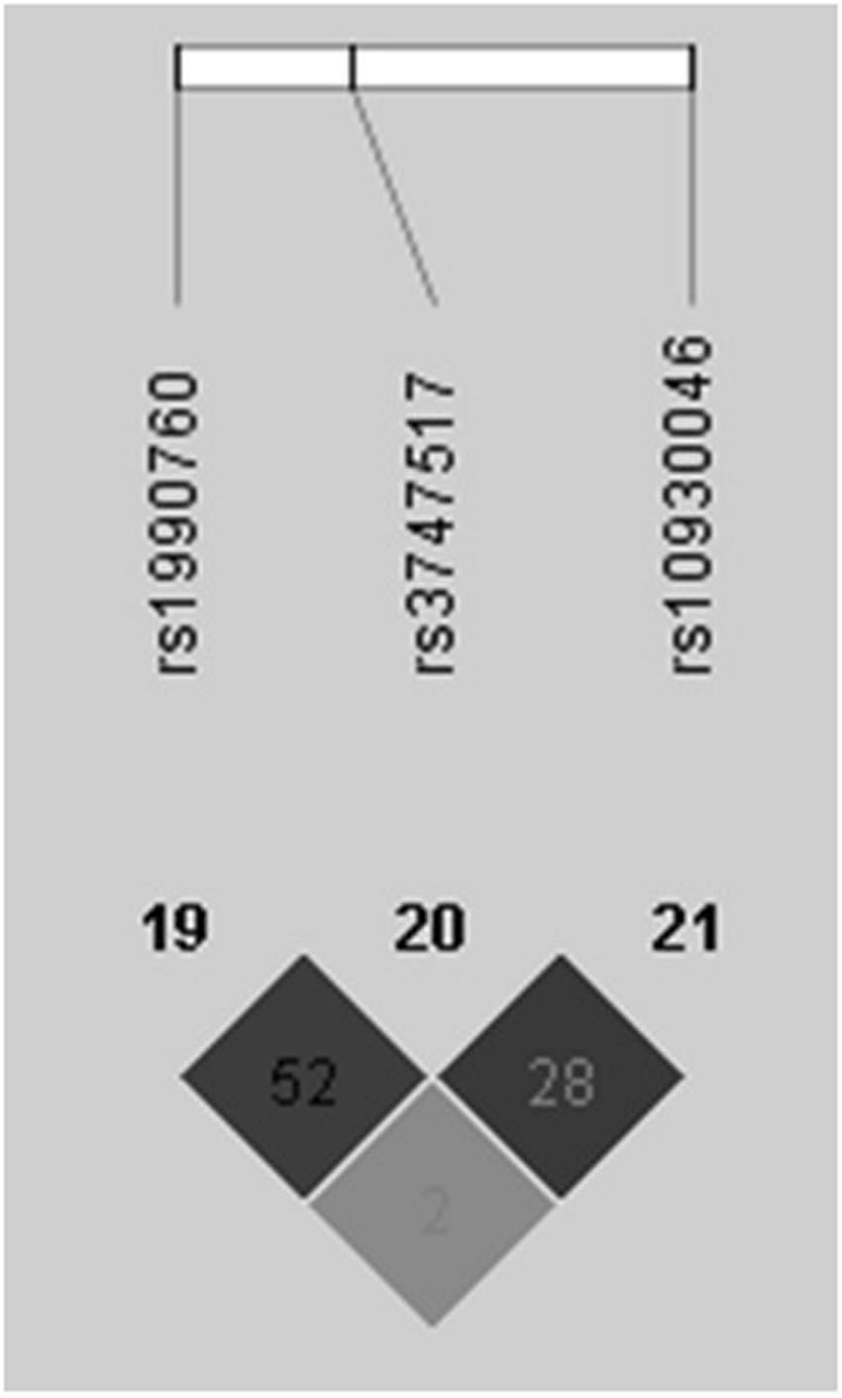
Linkage disequilibrium for SNPs of IFIH1 in 1059 individuals. The linkage disequilibrium plot shows r^2^ values between rs1990760, rs3747517 and rs10930046. There was not very strong LD between rs1990760 and rs3747517 (r^2^ = 0.52).

In our study, a significant positive association was observed between SLE susceptibility and variants of IFIH1 (rs1990760 and rs3747517), suggesting that IFIH1 genetic variants might have potential effects on increasing the risk of SLE in Chinese Han population. Similar to our findings, as Fig. 3 showed, rs1990760 and rs3747517 were not very strong in linkage disequilibrium (r^2^ = 0.52), the IFIH1 risk haplotype was also found to be in a moderate linkage disequilibrium^10^. Individuals carrying the risk haplotype exhibited an increased number of IFN signature genes compared to individuals carrying the non-risk haplotype^10^. What is more, significant positive association was observed between rs1990760 and the serum levels of IL-18, granzyme B as well as anti-dsDNA. We found that the carriers with TT genotype at rs1990760 had significant lower IL-18 and granzyme B concentration when compared to patients carrying CC and CT genotypes. The carriers with CT genotype at rs1990760 had significantly higher positive frequency of anti-dsDNA compared with CC or TT genotype patients of SLE in the Chinese Han population. Consistent with a previous study among African-American, European-American and Hispanic-American population^16^, we found that there was a trend of higher frequency for the presence of anti-dsDNA for rs1990760 heterozygote CT in Chinese SLE patients.

As a member of the IL-1 family, IL-18 is a cytokine promptly secreted from cells including antigen presenting cells (APCs) and T cells, which can raise the imbalance in Th17 differentiation pathway though directly stimulating Th1 cells^26^ and leading to the Th2 immune response^27^. It is closely related to SLE through leading to severe tissue inflammation and autoimmunity. This IFN–inducing factor is significantly higher in SLE patients and it can act as a vital factor in the pathogenesis^28,29^. That is because, caspase-1 splits proIL-18 into biologically active IL-18 form, and IL-18 can be activated by inflammasomes in SLE patients^30^. In addition, IL-18 participates in the dysfunction of endothelial cells and has impaired function of vascular repair in SLE^31^.

Elevated granzyme B might reflect a state of hyper-inflammation in chronic inflammation^32,33^. As the most characterized serine protease, granzyme B produced by T cells plays an essential role in acquired immune responses^34–36^, and the presentation of autoantigens through aiding in the generation of antigenic epitopes and stimulating autoreactive T cells^37–39^. In SLE patients, effector lymphocytes expressing granzyme B are present at high level, which is correlated with disease activity^37^. Through contributing to the pathogenesis of SLE/LN, the increased level of granzyme B in the serum as well as kidney of SLE patients was correlated with poor prognosis in LN^37^. Interestingly, granzyme B also had function in activating IL-18 through cleaving pro-IL-18 into its active form^40^, which might partly explain the simultaneous elevation.

The rs1990760 risk allele is predicted to result in an amino acid change from alanine to threonine at codon 946 within IFIH1^7,10,16^. While multiple studies have replicated and expanded the range of disorders associated with the IFIH1 autoimmune risk variant (rs1990760), its mechanistic impact(s) on immune tolerance *in vitro* and *in vivo* remains unknown.

A recent report found peripheral blood mononuclear cells (PBMCs) from healthy donors with the risk T allele at rs1990760 exhibit increased ligand-triggered IFN-I production. IFIH1 rs1990760 leads to an increased IFN state through recognition of self-RNAs and that this may increase risk for autoimmune disease^10^.

However, previous study demonstrated that the rs1990760 T allele was associated with lower serum IFN-α levels in SLE patients who had anti-dsDNA antibodies (p = 0.0026)^16^, suggesting that the IFIH1 rs1990760 T allele was associated with increased IFN-induced gene expression in PBMCs and increased IFN sensitivity but lower serum IFN-α levels in anti-dsDNA–positive SLE patients^16,41^. Similar to our findings, we found that the patients with risk TT genotype had lower serum IL-18 and granzyme B levels when compared to patients carrying CC and CT genotypes. In this study, as expected, these data suggested that the patients with risk T allele of IFIH1 (rs1990760) might provides more innate immune defects and indicate more autoimmune damage.

In summary, our study found a significant positive association between two SNPs (rs1990760 and rs3747517) in IFIH1 and SLE susceptibility. Our results first indicated that the SLE patients with TT genotype at rs1990760 had significant lower serum IL-18 and granzyme B than patients carrying CC and CT genotypes. The SLE patients with CT genotype at rs1990760 had significant higher positive frequency of anti-dsDNA than patients with CC or TT genotype in the Chinese Han population. Based on our study, we suggest that the IFIH1 rs1990760 risk T allele was associated with increased susceptibility of SLE as well as increased anti-dsDNA–positive of disease but lower serum levels of IL-18 and granzyme B in SLE patients. However, the future research should be conducted with a larger sample population and should include the mechanism study of IFIH genetic variants on the pathogenesis of SLE *in vitro* and *in vivo*.

## References

[1] Arkatkar T (2017). B cell-derived IL-6 initiates spontaneous germinal center formation during systemic autoimmunity. The Journal of experimental medicine.

[2] Rossi EA (2013). Trogocytosis of multiple B-cell surface markers by CD22 targeting with epratuzumab. Blood.

[3] Long H, Yin H, Wang L, Gershwin ME, Lu Q (2016). The critical role of epigenetics in systemic lupus erythematosus and autoimmunity. Journal of autoimmunity.

[4] Choubey D, Panchanathan R (2017). Absent in Melanoma 2 proteins in SLE. Clinical immunology (Orlando, Fla.).

[5] Menon M, Blair PA, Isenberg DA, Mauri C (2016). A Regulatory Feedback between Plasmacytoid Dendritic Cells and Regulatory B Cells Is Aberrant in Systemic Lupus Erythematosus. Immunity.

[6] Price JV (2013). Protein microarray analysis reveals BAFF-binding autoantibodies in systemic lupus erythematosus. The Journal of clinical investigation.

[7] Molineros JE (2013). Admixture mapping in lupus identifies multiple functional variants within IFIH1 associated with apoptosis, inflammation, and autoantibody production. PLoS genetics.

[8] Ferreira RC (2010). Association of IFIH1 and other autoimmunity risk alleles with selective IgA deficiency. Nature genetics.

[9] Kato H (2006). Differential roles of MDA5 and RIG-I helicases in the recognition of RNA viruses. Nature.

[10] Gorman JA (2017). The A946T variant of the RNA sensor IFIH1 mediates an interferon program that limits viral infection but increases the risk for autoimmunity. Nature immunology.

[11] Van Eyck L (2015). Brief Report: IFIH1 Mutation Causes Systemic Lupus Erythematosus With Selective IgA Deficiency. Arthritis & rheumatology (Hoboken, N.J.).

[12] Cunninghame Graham DS (2011). Association of NCF2, IKZF1, IRF8, IFIH1, and TYK2 with systemic lupus erythematosus. PLoS genetics.

[13] Zouk H, Marchand L, Polychronakos C (2010). Study of transcriptional effects in Cis at the IFIH1 locus. PloS one.

[14] Gateva V (2009). A large-scale replication study identifies TNIP1, PRDM1, JAZF1, UHRF1BP1 and IL10 as risk loci for systemic lupus erythematosus. Nature genetics.

[15] Downes, K. *et al*. Reduced expression of IFIH1 is protective for type 1 diabetes. *PloS one* **5**, 10.1371/journal.pone.0012646 (2010). 10.1371/journal.pone.0012646 PMC2936573 20844740

[16] Robinson T (2011). Autoimmune disease risk variant of IFIH1 is associated with increased sensitivity to IFN-alpha and serologic autoimmunity in lupus patients. Journal of immunology (Baltimore, Md.: 1950).

[17] Hochberg, M. C. Updating the American College of Rheumatology revised criteria for the classification of systemic lupus erythematosus. *Arthritis and rheumatism* **40**, 1725, 10.1002/1529-0131(199709)40:9&lt;1725::AID-ART29&gt;3.0.CO;2-Y (1997). 10.1002/art.1780400928 9324032

[18] Barrett JC, Fry B, Maller J, Daly MJ (2005). Haploview: analysis and visualization of LD and haplotype maps. Bioinformatics (Oxford, England).

[19] Chiba A (2017). Activation status of mucosal-associated invariant T cells reflects disease activity and pathology of systemic lupus erythematosus. Arthritis research & therapy.

[20] Silva, J. A. *et al*. Association of interferon-induced helicase C domain (IFIH1) gene polymorphisms with systemic lupus erythematosus and a relevant updated meta-analysis. *Genetics and molecular research: GMR* **15**, 10.4238/gmr15048008 (2016). 10.4238/gmr15048008 27813554

[21] Enevold C (2014). Genetic polymorphisms of dsRNA ligating pattern recognition receptors TLR3, MDA5, and RIG-I. Association with systemic lupus erythematosus and clinical phenotypes. Rheumatology international.

[22] Cen H (2013). Association of IFIH1 rs1990760 polymorphism with susceptibility to autoimmune diseases: a meta-analysis. Autoimmunity.

[23] Cen H (2013). Association study of IFIH1rs1990760 polymorphism with systemic lupus erythematosus in a Chinese population. Inflammation.

[24] Chen G (2012). Genetic variants in IFIH1 play opposite roles in the pathogenesis of psoriasis and chronic periodontitis. International journal of immunogenetics.

[25] Vergara C, Thio CL, Thomas D, Duggal P (2016). Polymorphisms in melanoma differentiation-associated gene 5 are not associated with clearance of hepatitis C virus in a European American population. Hepatology (Baltimore, Md.).

[26] Pham OH (2017). T cell expression of IL-18R and DR3 is essential for non-cognate stimulation of Th1 cells and optimal clearance of intracellular bacteria. PLoS pathogens.

[27] Fujikura D (2018). Aureobasidium pullulans-cultured fluid induces IL-18 production, leading to Th1-polarization during influenza A virus infection. Journal of biochemistry.

[28] Jafari-Nakhjavani MR, Abedi-Azar S, Nejati B (2016). Correlation of plasma interleukin-18 concentration and severity of renal involvement and disease activity in systemic lupus erythematosus. Journal of nephropathology.

[29] Gross O, Thomas CJ, Guarda G, Tschopp J (2011). The inflammasome: an integrated view. Immunological reviews.

[30] Kahlenberg JM, Kaplan MJ (2014). The inflammasome and lupus: another innate immune mechanism contributing to disease pathogenesis?. Current opinion in rheumatology.

[31] Yang CA, Chiang BL (2015). Inflammasomes and human autoimmunity: A comprehensive review. Journal of autoimmunity.

[32] Anthony DA, Andrews DM, Watt SV, Trapani JA, Smyth MJ (2010). Functional dissection of the granzyme family: cell death and inflammation. Immunological reviews.

[33] Lauw FN (2000). Soluble granzymes are released during human endotoxemia and in patients with severe infection due to gram-negative bacteria. The Journal of infectious diseases.

[34] Wang J (2018). Cytotoxic T cell responses to Streptococcus are associated with improved prognosis of oral squamous cell carcinoma. Experimental cell research.

[35] Garcia-Laorden MI (2016). Granzymes A and B Regulate the Local Inflammatory Response during Klebsiella pneumoniae Pneumonia. Journal of innate immunity.

[36] Piva L (2012). Cutting edge: Clec9A+ dendritic cells mediate the development of experimental cerebral malaria. Journal of immunology (Baltimore, Md.: 1950).

[37] Kok HM (2017). Systemic and local granzyme B levels are associated with disease activity, kidney damage and interferon signature in systemic lupus erythematosus. Rheumatology (Oxford, England).

[38] Sozzani S, Del Prete A, Bosisio D (2017). Dendritic cell recruitment and activation in autoimmunity. Journal of autoimmunity.

[39] Newby BN (2017). Type 1 Interferons Potentiate Human CD8(+) T-Cell Cytotoxicity Through a STAT4- and Granzyme B-Dependent Pathway. Diabetes.

[40] Omoto Y (2006). Human mast cell chymase cleaves pro-IL-18 and generates a novel and biologically active IL-18 fragment. Journal of immunology (Baltimore, Md.: 1950).

[41] Weckerle CE (2011). Network analysis of associations between serum interferon-alpha activity, autoantibodies, and clinical features in systemic lupus erythematosus. Arthritis and rheumatism.

